# Supplementary material for: An optimized CRISPR/Cas9 approach for precise genome editing in neurons
Source: eLife. 2021 Mar 10;10:e65202. doi: 10.7554/eLife.65202 (PMC7946428; doi:10.7554/eLife.65202)
Supplement: Supplementary file 1. [file elife-65202-supp1.docx]

**Table supplement**

**Table supplement 1. Location and sequences of guides**

| **Gene** | **guide1** | **PAM** | **Strand** | **Score On/Off** | **guide2** | **PAM** | **Strand** | **Score On/Off** | **guide1's location** | **guide2's location** |
| --- | --- | --- | --- | --- | --- | --- | --- | --- | --- | --- |
| **MOUSE** |  |  |  |  |  |  |  |  |  |  |
| Gria1 | GGGAAGACCAAATCTATGGT | TGG | + | 56/75 | TTAGCAATGGAACACCAGGA | AGG | + | 67/57 | 5'-utr(exon1) | intron 1-2 |
| Gria2 | AACAGCCACCAGCTAAACCT | GGG | + | 69/65 | TATGCCTTTTGACACAATAG | AGG | + | 64/63 | 5'-utr(exon1) | intron 1-2 |
| Gria3 | GCGAGCGAGAGCAAGTTGAG | GGG | + | 68/80 | CTGCAAGAGGCTAAGAGTCG | GGG | + | 67/72 | 5'-utr(exon1) | intron 1-2 |
| Grin1 | GCGCTGCTCGAACACCCGCG | CGG | - | 69/93 | TCTGTTCCATCTAGTCAGTG | TGG | + | 71/64 | 5'-utr(exon1) | intron 1-2 |
| Grin2A | GCTCGGCTTGGACTGATACG | TGG | + | 63/93 | ACACCGAGCAATTCTCACAA | AGG | - | 71/70 | intron 2-3 | intron 3-4 |
| Grin2B | AAGAACCTCGTCTCCGTCTG | CGG | - | 64/89 | CATTGAATGACCCTTTCACA | GGG | + | 65/60 | intron 2-3 | intron 3-4 |
| Gphn | CGACCCCGAGTCCGGCCGGG | AGG | - | 63/89 | GAGAAACCTCCAGCAAGTCG | CGG | - | 67/78 | 5'-utr(exon1) | intron 1-2 |
| Gabrb2 | ggcgtaccaaaacatcaaag | GGG | - | 77/42 | agtctggtcacactctaagg | GGG | - | 69/65 | 5'-utr(exon2) | intron 3-4 |
|  |  |  |  |  |  |  |  |  |  |  |
| **RAT** |  |  |  |  |  |  |  |  |  |  |
| Gria1 | GGGAAGACCAAATCTATGGT | TGG | + | 70/63 | CATGCTCGTCAAATGCCACG | AGG | + | 72/59 | 5'-utr(exon1) | intron 1-2 |
| Gria2 | ATATCGACCTCACAATGCAG | AGG | + | 61/64 | TATGCCTTTTGACACAATAG | CGG | + | 52/86 | 5'-utr(exon1) | intron 1-2 |

**On:** on-target score

**Off:** off-target score
