# Supplementary material for: An optimized CRISPR/Cas9 approach for precise genome editing in neurons
Source: eLife. 2021 Mar 10;10:e65202. doi: 10.7554/eLife.65202 (PMC7946428; doi:10.7554/eLife.65202)
Supplement: Supplementary file 2. [file elife-65202-supp2.docx]

**Table supplement 2. PCR primers for TKIT donors**

| **SEP-mGluA1** |  |
| --- | --- |
| A1_fwd | GGCCTTCCTGGTGTTCCATTGCTAAGGTTGGACCAGGGCTTCT |
| A1_rev | GTGGGAAGACCAAATCTATGGTTGGTGGTGTTCCATTGCTAAGC |
|  |  |
| **SEP-mGluA2** |  |
| A2_fwd | GGCCTCTATTGTGTCAAAAGGCATACCTGGGAGATAAGGATTC |
| A2_rev | GGAACAGCCACCAGCTAAACCTGGGTTGTGTCAAAAGGCATAGT |
|  |  |
| **SEP-mGluA3** |  |
| A3.up_fwd | tccgcgcacatttccccgactcgagaattcccccatactcgtcagttctttgcAGGGGGTGTAAGAGCCGG |
| A3.up_rev | agccaccgccaccGGGGAATCCTCCGTGAGAATG |
| A3.SEP_fwd | cggaggattccccGGTGGCGGTGGCTCTAGAAG |
| A3.SEP_rev | tgttggggaatccGGATCCGCCACCGCCTGA |
| A3.dn_fwd | cggtggcggatccGGATTCCCCAACACCATC |
| A3.dn_rev | ctcgctgtatcgctcgagggatccgaattcgcgagcgagagcaagttgaggggAGCTCAGGTCTCCAATATC |
|  |  |
| **SEP-mNR1** |  |
| N1.up_fwd | tccgcgcacatttccccgactcgagaattcccacactgactagatggaacagaCCAATACGCTTCAGCACCTCGGACAG |
| N1.up_rev | agccaccgccaccGGCAGCGCGGGCGAAGGA |
| N1.SEP_fwd | cgcccgcgctgccGGTGGCGGTGGCTCTAGAAG |
| N1.SEP_rev | cgcaggcagcgcgGGATCCGCCACCGCCTGA |
| N1.dn_fwd | cggtggcggatccCGCGCTGCCTGCGACCCC |
| N1.dn_rev | ctcgctgtatcgctcgagggatccgaattcccgcgcgggtgttcgagcagcgcTCACCAATGATAGTCACATGACAGCACACATGGTCC |
|  |  |
| **SEP-mNR2A** |  |
| 2A.up_fwd | tccgcgcacatttccccgactcgagaattcacaccgagcaattctcacaaaggAGATGGACATGGGTCGAAGAATGTGG |
| 2A.up_rev | agccaccgccaccCTTCTCCGCCGCCGCGTT |
| 2A.SEP_fwd | ggcggcggagaagGGTGGCGGTGGCTCTAGAAG |
| 2A.SEP_rev | gagtacccttctcGGATCCGCCACCGCCTGA |
| 2A.dn_fwd | cggtggcggatccGAGAAGGGTACTCCAGCG |
| 2A.dn_rev | ctcgctgtatcgctcgagggatccgaattcgctcggcttggactgatacgtggTCAGAGGCCACTTAACCTG |
|  |  |
| **SEP-mNR2B** |  |
| 2B.up_fwd | tccgcgcacatttccccgactcgagaattcccctgtgaaagggtcattcaatgGATTTTCTAGCATTCAGACACTTC |
| 2B.up_rev | agccaccgccaccGCTCTTTTGGGAACGAGC |
| 2B.SEP_fwd | ttcccaaaagagcGGTGGCGGTGGCTCTAGAAG |
| 2B.SEP_rev | gggcgctcttttgGGATCCGCCACCGCCTGA |
| 2B.dn_fwd | cggtggcggatccCAAAAGAGCGCCCCCAGC |
| 2B.dn_rev | ctcgctgtatcgctcgagggatccgaattcccgcagacggagacgaggttcttCCTGAATGGGTCACGACC |
|  |  |
| **SEP-mbeta2** |  |
| Gb2.up_fwd | tccgcgcacatttccccgactcgagaattcagtctggtcacactctaagggggGTGCGCATGCGCTCCACA |
| Gb2.up_rev | agccaccgccaccATTGACACTAAAGAAAAAAATGACAATAACCAGGAATGAATAAAGAAG |
| Gb2.SEP_fwd | ctttagtgtcaatGGTGGCGGTGGCTCTAGAAG |
| Gb2.SEP_rev | ggtcattgacactGGATCCGCCACCGCCTGA |
| Gb2.dn_fwd | cggtggcggatccAGTGTCAATGACCCTAGTAATATG |
| Gb2.dn_rev | ctcgctgtatcgctcgagggatccgaattccccctttgatgttttggtacgccTCTTCTTGAAAGACCTCTC |
|  |  |
| **GFP-Gephyrin** |  |
| gep.up_fwd | ctcgctgtatcgctcgagggatccgaattcgagaaacctccagcaagtcgcggCGCGGCCCGACTCCGCCC |
| gep.up_rev | cccttgctcactctagaCATGTTTCCCAGCGCAGTCACCGCAC |
| GFP_fwd | gctgggaaacatgtctagaGTGAGCAAGGGCGAGGAG |
| GFP_rev2 | cgagccaccgccaagCTTGTACAGCTCGTCCATGC |
| gep.dn_fwd | cgagctgtacaagcttggcggtggctcgggcggaggtgggtcaGCGACCGAGGGAATGATC |
| gep.dn_rev | tccgcgcacatttccccgactcgagaattccctcccggccggactcggggtcgCGGATAGTAGCCGTTGCG |
|  |  |
| **SEP-rGluA1** |  |
| rA1.upSEP_fwd | gcacatttccccgactcgagcctcgtggcatttgacgagcatgGGTTGGACCAGGGCTTCTTTTTCG |
| rA1.upSEP_rev | ggaaattggcGGATCCGCCACCGCCTGA |
| rA1.dn_fwd | tggcggatccGCCAATTTCCCCAACAATATC |
| rA1.dn_rev | aggaagcggaagagcgcccagggaagaccaaatctatggttggGGCATTTGACGAGCATGAAAC |
|  |  |
| **SEP-rGluA2** |  |
| rA2.up_fwd | tccgcgcacatttccccgactcgagccgctattgtgtcaaaaggcataCAGAGGATCTAATTTGCTG |
| rA2.up_rev | cgccaccactagaTATGCTGTTAGAAGAGACAC |
| rA2.SEP_fwd | ttctaacagcataTCTAGTGGTGGCGGTGGC |
| rA2.SEP_rev | tctgtatgctgttGGATCCGCCACCGCCTGA |
| rA2.dn_fwd | cggtggcggatccAACAGCATACAGATAGGTAG |
| rA2.dn_rev | gagcgaggaagcggaagagcgcccaatatcgacctcacaatgcagaggTTGTGTCAAAAGGCATAC |
|  |  |
| **PCR primers for AAV constructs** |  |
| **SEP-GluA1 & SEP-GluA2** |  |
| donor_fwd | ccatcactaggggttcctgcggccgcACATTTCCCCGACTCGAG |
| donor_rev | agcaaaaggccagCGCTCGAGGGATCCGAATTC |
| 2guides_fwd | ccctcgagcgCTGGCCTTTTGCTCACATG |
| 2guides_rev | ccatcactaggggttcctgcggccgcTTATGTAACGGGTACCACC |
